# Addressing a systematic error correcting for free and mixed convection when measuring mean radiant temperature with globe thermometers

**DOI:** 10.1038/s41598-022-10172-5

**Published:** 2022-04-19

**Authors:** Eric Teitelbaum, Hayder Alsaad, Dorit Aviv, Alexander Kim, Conrad Voelker, Forrest Meggers, Jovan Pantelic

**Affiliations:** 1 CHAOS Laboratory, Princeton University, Princeton, NJ 08544 USA; 2 AIL Research, Inc., Hopewell, NJ 08525 USA; 3 Department of Building Physics, Bauhaus-University Weimar, Weimar, 99423 Germany; 4 Weitzman School of Design, University of Pennsylvania, Philadelphia, PA 19104 USA; 5 KU Leuven, Faculty of Bioscience Engineering, Department of Biosystems, Leuven, Belgium; 6 Well Living Lab, Inc., Rochester, MN 55902 USA

**Keywords:** Mechanical engineering, Characterization and analytical techniques

## Abstract

It is widely accepted that most people spend the majority of their lives indoors. Most individuals do not realize that while indoors, roughly half of heat exchange affecting their thermal comfort is in the form of thermal infrared radiation. We show that while researchers have been aware of its thermal comfort significance over the past century, systemic error has crept into the most common evaluation techniques, preventing adequate characterization of the radiant environment. Measuring and characterizing radiant heat transfer is a critical component of both building energy efficiency and occupant thermal comfort and productivity. Globe thermometers are typically used to measure mean radiant temperature (MRT), a commonly used metric for accounting for the radiant effects of an environment at a point in space. In this paper we extend previous field work to a controlled laboratory setting to (1) rigorously demonstrate that existing correction factors used in the American Society of Heating Ventilation and Air-conditioning Engineers (ASHRAE) Standard 55 or ISO7726 for using globe thermometers to quantify MRT are not sufficient; (2) develop a correction to improve the use of globe thermometers to address problems in the current standards; and (3) show that mean radiant temperature measured with ping-pong ball-sized globe thermometers is not reliable due to a stochastic convective bias. We also provide an analysis of the maximum precision of globe sensors themselves, a piece missing from the domain in contemporary literature.

## Introduction

We present a systematic analysis of a radiant heat sensor that also serves a cautionary tale of systematic error embedded in a measurement device when its utilization in research and practice becomes the unquestioned default. In this case, the recognition of the importance of radiant heat transfer in the heat balance of the human body in the early 1900’s presented the problem of measuring the phenomena, which initially required complex equipment and techniques. These were dramatically simplified with the introduction of the black globe thermometer as it could directly output changes in temperature caused by radiation, which could be correlated to represent radiant heat transfer effects. We have uncovered that while the initial use of the globe involved recognition of the important effects of convective exchange between it and air moving around it as it was radiatively heated or cooled, larger radiant fluxes start to generate significant buoyancy flows around the globe and have been systematically neglected in the decades since the globe thermometer has become standard, and the inherent device physics were subsequently disassociated from the tool itself. Presently, the most commonly used standards for measuring radiant effects in the United States completely neglect free convection and lead to systematic under-reporting of radiant heat effects. We detail the significance of this effect and its role in the ASHRAE and ISO standards, and we propose ways to improve and re-validate the operation of globe thermometers while also more clearly describing significant limitations that have been forgotten over the years as their use has become commonplace.

More specifically, mean radiant temperature is currently the most common metric used to describe the radiant heat transfer experienced by a human body in space. This has its roots in characterizing thermal exchanges, mainly blackbody emission of infrared heat, to people indoors beginning in the 1930’s^1^ in the area of so-called "industrial hygiene"^2^ for workers. Later, it shifted to the study of thermal comfort as heating and cooling systems became more advanced^3–6^. With Fanger’s seminal thermal comfort work in the 1970’s^4^, Mean Radiant Temperature (MRT) became an integral component of thermal comfort calculations and remains part of the contemporary evolution of thermal comfort models such as adaptive comfort^7^. More recently, the fields of outdoor heat analysis, urban heat island impacts, and heat stress evaluation have begun using MRT to represent radiant heat experienced, which includes much larger potential inputs of shortwave solar radiation^8^, and this has now translated to new considerations of solar impacts on indoor radiant heat^9^. In a recent review we summarized the history of MRT and some of the challenges surrounding its interpretation^10^. While traditionally, outdoor and indoor thermal comfort are somewhat separate domains with separate comfort models, little thought has been given to the magnitude of free convection effects on globe thermometers indoors since despite larger radiative forcing in outdoor environments, higher air speeds produce high-fidelity results in outdoor settings^8^. However there remains uncertainty about the best globe thermometer construction methods, correction factors, and mean radiant temperature definitions^11^.

One challenge of the use of MRT to describe radiant heat transfer has been its measurement. Originally, radiant heat transfer was understood with devices that were complex and expensive^1,12^. In 1932, Vernon described how a simple blackened globe (a common spherical 6 inch (150 mm) copper float painted black) would come to equilibrium with the radiant conditions, and the mean radiant temperature could be calculated directly from that one measurement^13^. However, early researchers were cognizant of the fact that a thermometer only ever measures its own temperature, and great care was given to estimate the acceptable measurement ranges. In fact, there is much discussion in the literature about free convection’s effects on globe thermometers prior to the 1970s^1,14^.

The black globe greatly simplified the measurement of MRT, but the method required an important calibration against the movement of air around the globe as first described by Bedford and Warner in 1934^1^. Bedford and Warner confirmed the $\sqrt{V}$ relationship of air velocity to convection and validated a first correction of globe temperature to MRT using these values^1^. This enabled the simple method of inserting a thermometer inside a black globe and determining the MRT based on the measurement and the air speed around the globe.

Further development of measurement devices were focused on the reduction of size that lead to the suggestion by Humphreys in 1977^15^ and validation by de Dear 1988^16^ that the original 150 mm black globe can be reduced to a 40 mm ping-pong ball globe thermometer. This result was widely accepted, and used for decades as the default MRT measurement method^17^ because the measurement system is simple and low-cost, enabling high-granularity measurements. The experiment conducted by de Dear^16^ assumed that a 150 mm black globe provides correct ground truth measurements. The proposed de Dear method gained popularity due to the fast response time and further simplification of sensor construction. Previous work on 40 mm sensors demonstrated fast response times but large errors. There was no further validation, for example, using surface temperature measurements and view factor calculations.

The ubiquitous use of the globe thermometer was caused by its simplicity, and it was enabled by international standards put in place normalizing its utilization. In our study, we focus on the two primary standards used in MRT measurements—ASHRAE standard 55^6^ and ISO7726^18^. The two methods are largely equivalent, however ISO7726 has a method for choosing between free and forced convection, while ASHRAE ignores the free convection component. When forced convection is selected, the two methods are identical. This paper proposes a unified method of dealing with the effects of both free and forced convection to accurately measure mean radiant temperature in the built environment. A detailed description of the genesis of this model as well as a mathematical analysis for why the ISO7726 treatment of free convection is insufficient is provided in the Materials and Methods section.

In a previous experiment we first found significant systematic error in the correction of MRT with globes^19^ where measurement with globes showed maximum $2^{\circ }\hbox {C}$ difference between MRT and air temperature while pyrgeometer measurements showed $7^{\circ }\hbox {C}$ difference between MRT and air temperature. The experiment was largely uncontrolled and far from a rigorous experimental validation of new correction equations. This present paper offers the benefits of a rigorously controlled climate chamber to fully probe the ability of globe thermometers to measure mean radiant temperature when properly accounting for mixed convection.

### Historical context

To provide specific and quantified context to this paper within a building physics domain with a century of discovery, methodologies from relevant articles from the 1930s to 2020 were examined to determine the tools and techniques applied to calculate MRT, as well as the conclusions drawn from the measurements. In total, 54 articles were analyzed, with the most studies (17 papers) being published in the 2010s. To obtain relevant articles, first every study involving MRT or globe thermometers added to the ASHRAE Thermal Comfort Database I to produce the ASHRAE Thermal Comfort Database II was reviewed. Next, older publications were found by targeting specific developments in the field, such as the normalization of the ping-pong globe thermometer^16^ and the development of the PMV model^4^, and by reviewing the articles cited by those seminal studies. We have extensively reviewed the historical context of globe thermometer use for mean radiant temperature since the first use of the globe corrected by forced convection in 1934. The historical context provides insights into how the systematic lack of consideration for convective corrections evolved into standard practice producing in the systematic bias we present in this paper.

From the mid-to-late 1930s when globe thermometers and the concept of a ‘mean radiant temperature’ were new through the early 1960s, there were many papers that used a similar method as the one included in this paper—a thermopile in conjunction with globe thermometers—to validate measurements^1,12,13,20,21^. It was common to use a silvered globe to measure convective heat losses as a method to provide greater certainty to the mean radiant temperature as extrapolated from the black globe.

A salient detail is that comfort metrics were in their infancy. Effective temperature, operative temperature, and wet bulb globe temperature scales were all in their nascency. The concept of ‘equivalent temperatures’ was a major conceptual breakthrough, emerging from Yaglou’s research in the 1920s^3^ and extended by Koch^21^ and others into the 1960s to mean radiant temperature. The concept of abstracting heat transfer to equivalent environments required accurately monitoring environmental criteria, which required accurate measurement tools and devoting significant thought to measurement devices and techniques.

This paradigm of gathering accurate data for developing new standards to predict physiological responses in environments shifted starkly in the later 1960s when tools to ‘quickly’ assess environmental conditions became desirable. The goal became to evaluate environments in the context of the emerging standards developed with care in previous decades. Researchers proposed methods for generating quick measurements^22,23^, yet both of these authors discuss the large error (10%) and that small globes should be used for qualitative measurements only.

A follow up study in 1977^15^ claimed that a 40mm globe thermometer is optimal for assessing the warmth of a room for human comfort because the ratio of radiative heat to convective heat experienced by the globe is closer to a human than that of a larger globe. However, there is a large caveat that larger globes are still better for measuring the mean radiant temperature because this proportion is skewed more towards radiation. This argumentation again invokes the measurement paradigm: fidelity to environment or fidelity to comfort models.

A contemporary review of thermal comfort methods^24^ points out the trend of first measuring a single environmental factor precisely, and then creating an index from the combination of several factors. This was a prevalent research trend in the thermal comfort domain. This trend continued to repeat itself, into the subsequent decades.

With Fanger’s groundbreaking PMV/PPD comfort framework^4^ introduced in the 1970s, there is a transition in thermal comfort research toward using globes to produce an input to a comfort framework rather than for a fundamental environmental assessment. Fanger’s comfort model was arguably so successful since it was able to simplify a multidimensional comfort landscape into a singular numerical output that could be used for design.

With the desire to begin using these more manageable metrics as opposed to raw physical parameters comes methodological assumptions. The 1970s PMV/PPD literature includes many publications with a mean radiant temperature equal to air temperature assumption^25–29^. While these assumptions may be valid for the respective operating regimes, we believe it is indicative of a trend of shifting away from measurement fidelity to providing metrics for models explicitly.

While this is a small subset of the PMV literature, other studies from this time period that did not make this equivalence relied on globe thermometer relationships that were rarely revisited. One paper in particular^30^ presents a novel correction equation, but this and others surveyed^31,32^ explicitly are seeking a quick means of surveying the thermal environment, thereby choosing a small ping pong ball. Humphreys^32^ even states that the ping pong ball is a convenient tool for measuring the warmth of a room when the air movement is slight. In these environments it is even more important to know precisely the radiant temperature since radiative heat transfer dominates the physiological exchange^33^.

#### Contemporary work and impacts

Into the 1980s through to today when adaptive comfort, personal comfort, and other empirically driven comfort analyses founded on computationally intensive analyses begin to dominate over Fanger’s heat balance model, a trend emerges of overconfidence in the error of mean radiant temperature measurements. In a survey of papers that comprise the ASHRAE thermal comfort databases I and II from 1997 through to 2020^34–47^, there is an average error of 0.70 $^{\circ }$C reported for the globe thermometer over the 14 studies with reported errors, which 12 out of 14 times is for a 40 mm or smaller sensor.

In addition to the analysis presented in this paper, ISO7726^18^ points out that values within 5 $^{\circ }$C are impractical to achieve with standard globe thermometers, calling into question the fidelity of modern comfort models with respect to true values for mean radiant temperature. In fact, the wording from the standard for a 2 $^{\circ }$C error is as follows: “These levels are difficult or even impossible to achieve in certain cases with the equipment normally available/ When they cannot be achieved, indicate the actual measuring precision".

**Figure 1:**
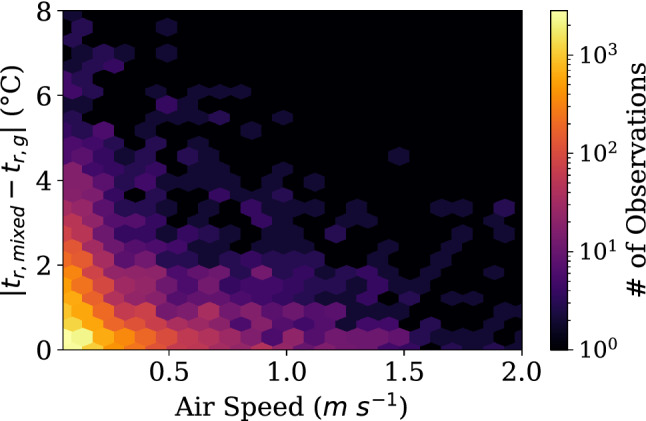
Distribution of potential error in $t_r$ reported in the ASHRAE Thermal comfort database II when comparing the reported $t_r$ compared to the $t_r$ calculated when accounting for mixed convection as presented in this paper. The color at each location is determined by the measurement frequency.

In short, if mixed convection is properly accounted for according to the findings in this paper, Fig. 1 shows how much absolute error could exist in readings in the comfort database versus air speed. The average error, calculated as the absolute value of the presented value minus the mixed convection correction to account for both radiant heating and cooling in the same metric, is 0.63 ± 0.78 $^{\circ }$C. This is a statistically significant error for 21,861 datapoints. Comfort models based on this empirical evidence may need to reconsider the effects of radiant temperatures. Likewise, the ASHRAE thermal comfort standard 55 has a requirement that MRT must be measured within 1 $^{\circ }$C^6^. We have demonstrated that this is an unreasonable expectation for small globe thermometers, and reflects a systematic overconfidence of the acceptability of globe thermometer measurements^48^.

In a recent review paper of the ASHRAE comfort database the difference between radiant temperature and air temperature is systematically analyzed^17^. It is done so only considering the reported database values of 200,000 measurements across 6593 locations. It is a rigorous statistical review, but it does not consider that these values were derived from globes based often unknown air velocity conditions. Comparisons were made with controlled laboratory data and a few field test, and for the MRT measurements the reported uncertainty measured by a globe is +/$-$ 0.04 $^{\circ }$C. This is the resolution of the instrument, and again as we demonstrated in our historical analysis, fails to register the intrinsic error in the derivation of the result from the device. From their review there is an average absolute difference between air and radiant temperature of only 0.4 $^{\circ }$C, and a 5th and 95th percentile range of relative difference being from $-$0.4 $^{\circ }$C to 1.6 $^{\circ }$C. In response to our initial paper discussing globe error^19^, the authors stated that *because* their results showed only differences less than 2 $^{\circ }$C, that they were not in a regime where convection mattered. This is in fact inverting the causality we have found. We argue with our previous work and now with this more rigorous climate chamber data in this paper, that it is in fact *because* free convection is *not* considered in the ASHRAE standard that the dataset systematically underreports the separation of radiant temperature from the air temperature. Based on our analysis, the values that do approach higher difference are the ones whose real difference could be more than triple the value measured by a small globe. It is also noted that when air movement is below 0.2 m/s, the radiant temperature is back-calculated from the measured operative temperature and air temperature. Operative temperature simply assumes the radiant heat transfer coefficient is equivalent to the linearized radiant heat transfer coefficient. And in fact, there are many examples measuring the operative temperature in the literature^49,50^. This avoids completely neglecting free convection, but it also ignores advice from the seminal operative temperature paper^51^ which introduces operative temperature as a nonphysical, abstract parameter with no physical interpretation.

The challenge presented by the systematic error in radiant temperature derivation from globe thermometers is that it under represents the impact of radiant temperature. In work studying the relative impact of thermal comfort variables on comfort the same researchers that found MRT separation from air temperature to be small also reported how air temperature can predict comfort more accurately than when including a wider variety of comfort variables such as MRT and air movement^48^. But both MRT and air movement are fraught with the challenge of measuring complex air flows. While we agree that there are opportunities to simplify comfort analysis, the failure to perform may not be due to the failure of the variables to physically represent an impact, but a failure of the measurement techniques to accurately represent the variables.

It seems apparent from these results that there is a significant potential for systematic error in the the most ubiquitous method used to measure mean radiant temperature. Although this was very clear to the researchers who developed the first correlations for the tool in 1934, who said "By itself the globe thermometer is inadequate as an index of the thermal environment," since then, the standardization of the tool has led to standard practices that fail to incorporate a complete picture of the heat transfer intricacies of the device. In the worse case, the standard described by ASHRAE neglects the effect of free convection on the device altogether. In the ISO7726 standard, the free convection vs forced convection corrections fail to capture what is arguably the most common flow regime in indoor spaces: mixed convection.

## Results

**Figure 2:**
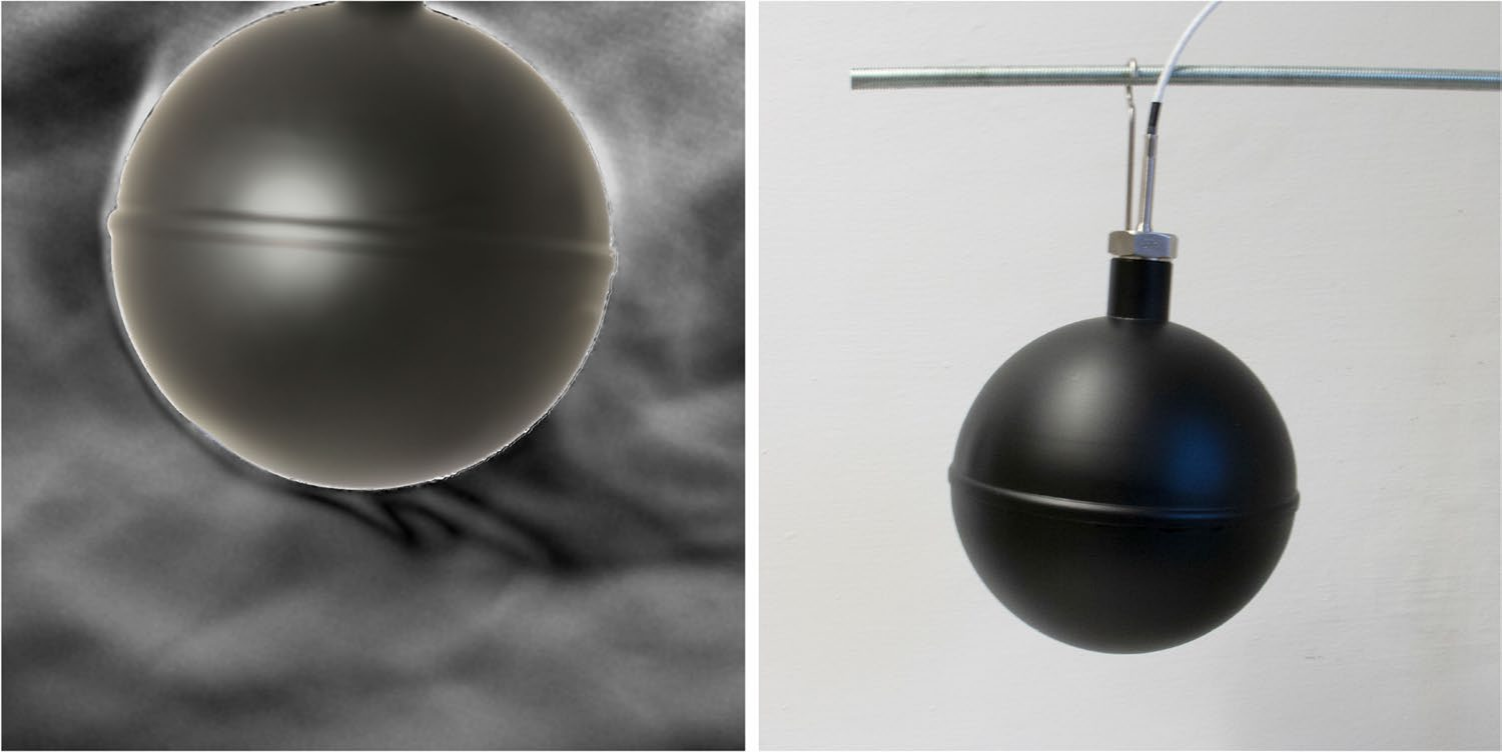
Background oriented schlieren imaging (left) of the globe thermometer setup (right) accentuating air density variation to illustrate air movement and convection around the globe. Dark lines at the bottom represent cool free convection separating from the globe.

When the surrounding temperatures, meaning surfaces that the globe thermometer radiatively exchanges heat with, differ on average from the air temperature, the surface temperature of the globe will differ from the air temperature. Hence, a convective flow develops around the globe. This effect is visible in Fig. 2, which shows the buoyancy-driven plume visualized using background oriented schlieren imaging^52^ when the separation between air temperature and surface temperature was 5.5 K. This image represents a mode of heat transfer occurring in many of the experiments; the subsequent results quantify a unified method of addressing both free and forced convection through a mixed convection parameter. All derivations of our proposed correction and the existing ISO/ASHRAE methods can be found in the *Materials and Methods* section. For clarity, Eq. 3 has been reproduced below in Eq. 1, showing where the convective heat transfer coefficient, $h_{cg}$ is inserted for all methods to provide the mean radiant temperature, $t_{r,g}$, from the air temperature, $t_a$, and globe temperature, $t_g$. Air speed, $v_a$, is the other measured parameter, and is used in the calculation of $h_{cg}$.

1 $$\begin{aligned} t_{r,g} = \root 4 \of {(t_g+273.15)^4-\frac{h_{cg}}{\varepsilon \sigma }(t_a-t_g)}-273.15 \end{aligned}$$

**Figure 3:**
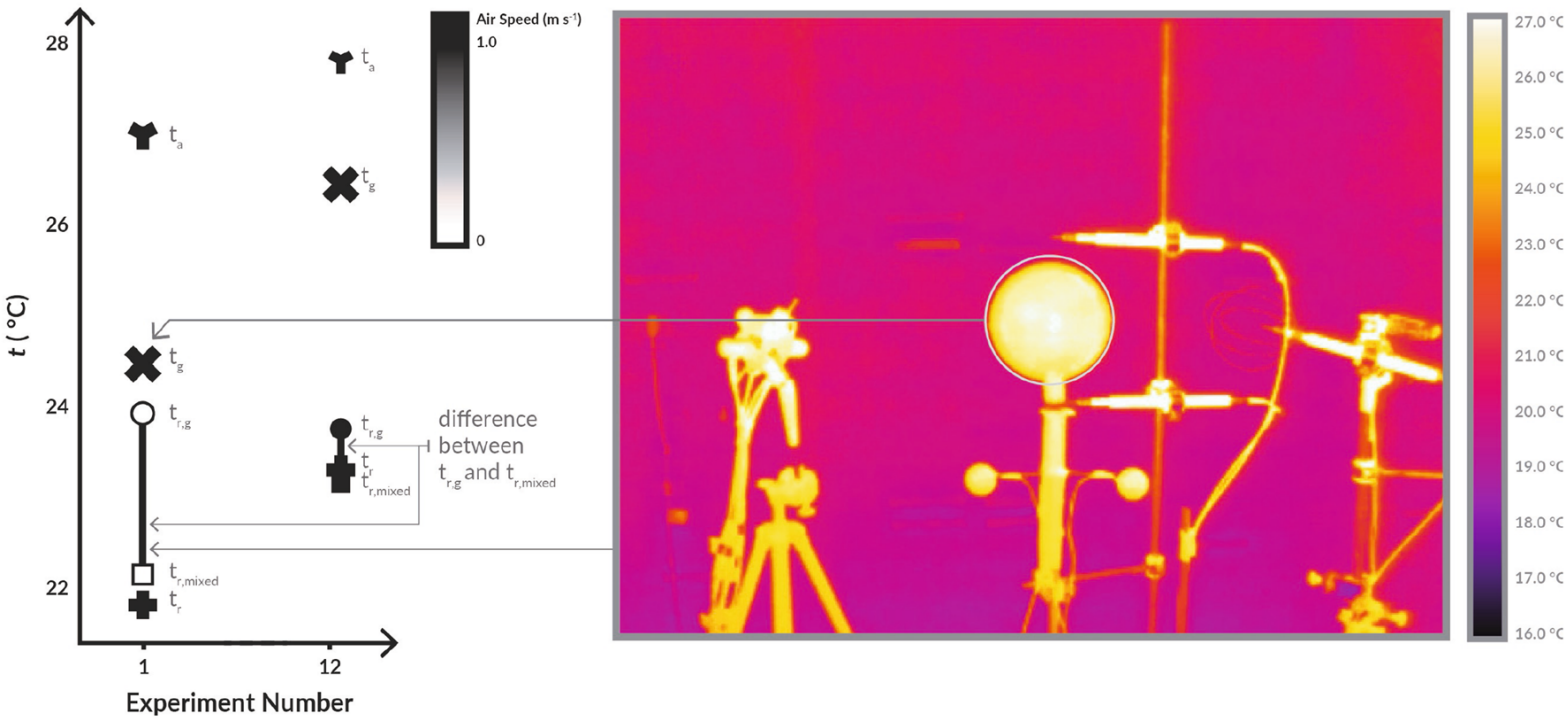
Explanation for how to interpret all data presented in Fig. 4 (left) and thermal image of experimental setup (right). The directly recorded physical measurements of air temperature ($t_a$ as **Y**), globe temperature ($t_g$ as **X**), and radiant temperature ($t_r$ as **+**). The calculated radiant temperatures determined from the globe are represented by the circle (current ISO7726 convection correction) and the square (proposed mixed convection correction). The difference between either $t_r$ calculated with the standard correction ($t_{r,g}$) or with our proposed correction factor ($t_{r,mixed}$) and the baseline measurement $t_r$ represents the impact of convection on the globe compared to a calibrated pyrgeometer measuring only the radiant heat transfer. The line represents the difference between the two corrections. The color (grayscale) fill of the shapes corresponds to the air speed during the measurement used for the forced and mixed convection correction calculations.

**Figure 4:**
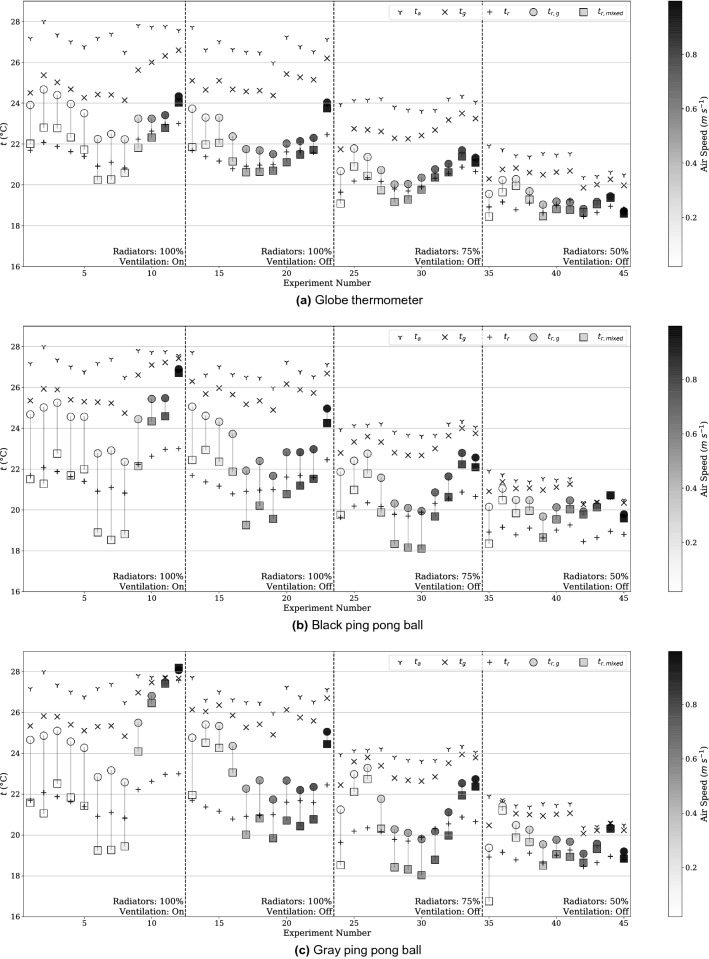
Data from all measurements for the standard globe (**a**), the black ping pong ball (**b**) and the gray ping pong ball (**c**) with each having a set of 4 climate chamber settings (Radiators/Ventilation settings) and a range air velocities within each of the chamber settings controlled by small axial fans. Data presented includes $t_a$, $t_g$, $t_r$, and a color mapped gradient corresponding to $v_a$. Additionally, the calculated values for $t_r$ are shown using the standard ASHRAE correction, $t_{r,g}$ and using the proposed mixed convection correction, $t_{r,mixed}$.

All raw data collected over the test cases is presented in Fig. 4, and an explanation for how to interpret the figure is presented in Fig. 3. The raw data collected with no formulas applied ($t_a$, $t_g$, $t_r$) are presented as **Y**, **X**, and **+**. The ISO7726 $t_r$ value is shown with a circle, and our mixed convection corrected $t_r$ is presented as a square. A line connects these two points, as this is the difference between the two corrections. $v_a$ is presented as a grayscale fill of the circle and square as it is an input into the convection corrections. Also in Fig. 3 is a thermal image of the experimental apparatus in the test chamber. Cool walls are visible compared to the temperature of the globe, which reduce the globe temperature relative to the air.

Figure 4 shows the full set of results for the standard globe and black and gray “ping pong" ball style globes. Three globes were measured in parallel for 45 settings generated by 4 general setpoint clusters in a climate chamber. The radiant panels and ventilation were set differently for each of the 4 sets to achieve a variety of separation of air temperature to radiant temperature. Within each of these series the measurements span a range of air speeds generated by controlled fans. Each measurement represents an average value over 30 minutes of assumed steady-state conditions, where no more than 0.5 K fluctuation was observed. There are a few trends. As air speed increases, $t_g$ approaches $t_a$, an expected trend since the magnitude of forced convection increases. However, this means the globe temperature can become much closer to the air temperature than it is to the radiant temperature, giving the correction factor a more significant role in determining the mean radiant temperature, magnifying any noise or error in the measurements of air temperature, $t_a$, globe temperature, $t_g$, and air speed $v_a$.

As expected, as air speed increases the ISO7726 model and our proposed mixed convection model converge, as seen in Fig. 4 where the line between the circle and squares shrinks as the velocity increases represented by the darker color. But as air speed increases there is not a trend towards convergence with the true pyrgeometer-measured $t_r$. Particularly for the smaller diameter ping pong balls, the data does not tend to converge at the true $t_r$ value even at high air speeds when natural convection effects that are not measured are small in comparison.

Further, the *n* parameter for mixed convection shown in Eq. (11) was varied until the deviation between $t_r$ and $t_{r,mixed}$ was minimized. For the standard globe, this value was $n=0.75$, and for ping pong balls was $n=0.62$. This parameter is used for all analyses.

The mixed convection correction clearly outperforms the ISO7726 at predicting the measured $t_r$ for the standard globe. It was consistently within 1 degree of the true value. The ISO7726 standard correction is consistently nearly double the error of the mixed convection correction and only outperforms it in 4 out of 45 measurements for the standard globe (not the same four cases with higher free convection versus foreced convection coeffciients used in the ISO7726 method). For the small globes there is generally poor performance by both correction models. At high air speeds, the small globe measurements nearly equalled the air temperature, making the derivation of the real radiant temperature based on $t_a-t_g$ impossible.

**Figure 5:**
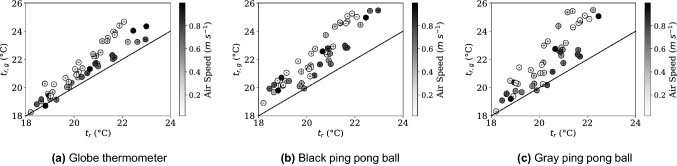
Mean radiant temperature, $t_{r,g}$ measurements using the ISO7726 correction factor for globe thermometers do not match the mean radiant temperature measurement produced using a 6 pyrgeometer sensor, $t_r$. The black line is $y=x$ representing a perfect correlation. Shading indicates air speed present for each data point, and error bars for the standard deviation of 30 minutes of data averages are included.

To put all data into relative comparison, globe thermometer data corrected using the ISO7726 method is shown on a scatter plot versus the pyrgeometer-measured $t_r$ values for the standard globe (Fig. 5a), black ping pong ball (Fig. 5b), and gray ping pong ball (Fig. 5c). The line $y=x$ is shown for comparison, as ideally all points fall on this line. This data represents the deviation from the expected value. The color of the points corresponds to the air speed and is shown on the color bar on the right of the image. While the largest deviation from $y=x$ occurs at low air speeds, there is significant variation for all air speeds. The data trends further from ideal for the smaller globes than the standard globe thermometer.

**Figure 6:**
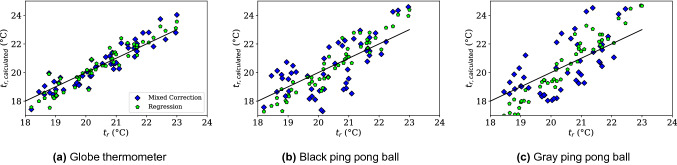
Bivariate linear regression using air speed and $t_a-t_g$ to predict $t_r$. The results of the regression for the standard globe thermometer overlap well with the empirical model, however the empirical model slightly outperforms ($R^2$=0.84 versus 0.92). For the ping pong balls, the regression distribution is tighter, however the slope is quite far off the $y=x$ line. $R^2$=0.65 and 0.37 for the black and gray ping pong balls, respectively.

The mixed convection corrected data is also plotted on a scatter plot in Fig. 6 as blue diamonds. In addition, a bivariate least squares regression was conducted correlating $t_a-t_g$ and $v_a$ with $t_r$ to see if a simple model could be derived independent of the physics-driven model, which is shown in Fig. 6 as green pentagons. For all globes it is clear that the empirical mixed convection model and the linear regression help remove the systematic bias shown in Fig. 5, where the standard ISO7726 globe correction systematically skews the radiant temperature towards air temperature. The results of the statistical regression for the standard globe thermometer overlap well with the physics based, calibrated empirical model, however the empirical model outperforms ($R^2$=0.84 versus 0.92 for the empirical model). For the ping pong balls, the regression distribution is better, however the slope is quite far off the $y=x$ line. $R^2$=0.65 and 0.37 for the black and gray ping pong balls, respectively. This statistically confirms the poorer performance of the small globes seen in Fig. 4. The regression form and coefficients can be found in Table 1.

**Table 1:** Regression coefficients and $R^2$ values for the standard globe, black, and gray ping pong balls. The form of the regression equation is $t_r$ $(^{\circ }C)$ $= c_1v_a + c_2(t_a-t_g)+b$.

	Black 150 mm	Black 40 mm	Gray 40 mm
$c_1$	2.0234	1.4308	0.3659
$c_2$	2.3186	2.3751	1.4877
*b*	$-$0.2498	1.4298	2.6881
$R^2$	0.84	0.65	0.38

**Figure 7:**
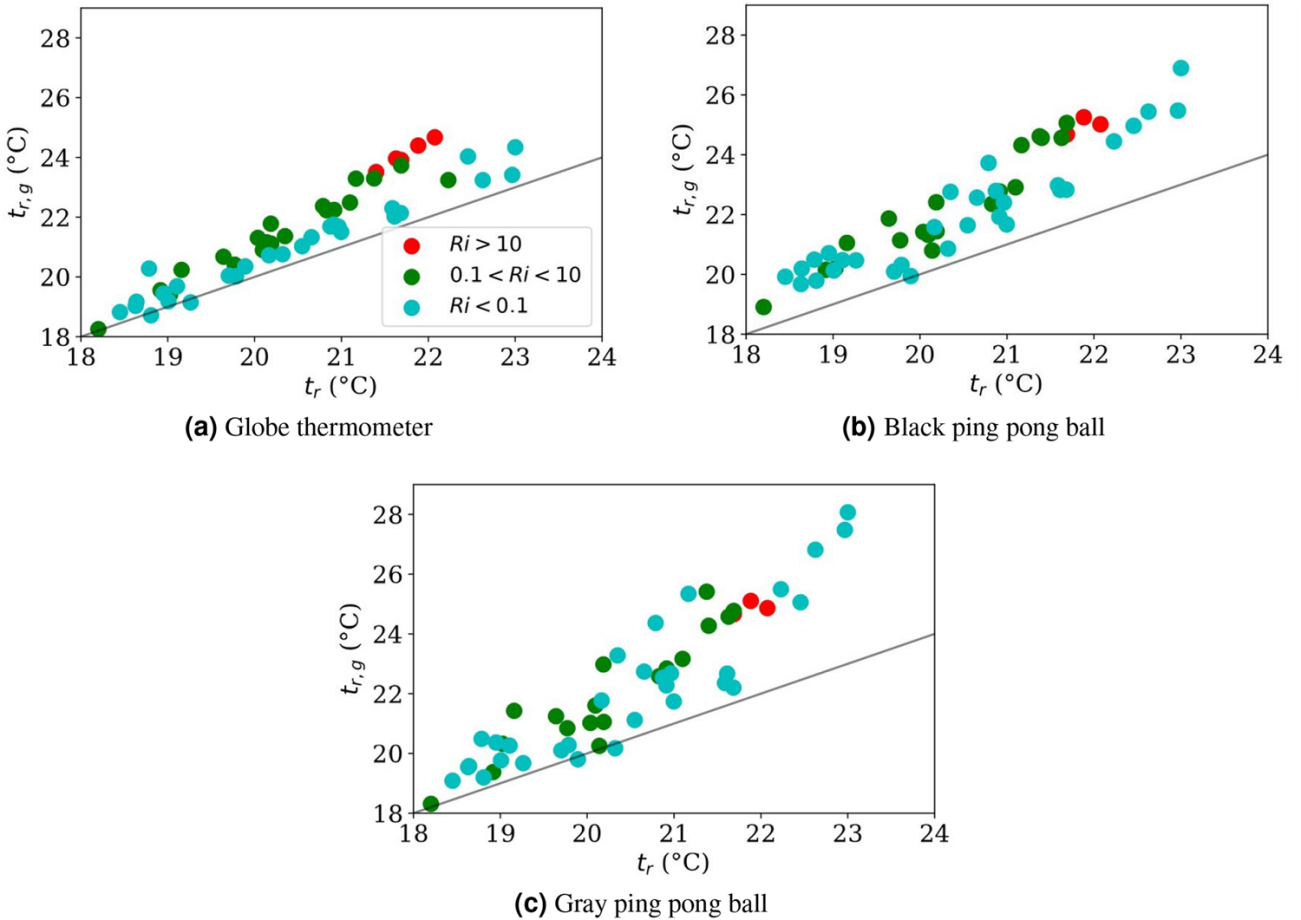
Coloring the datapoints from Fig. 5 based in *Ri*. Clustering is apparent for the standard globe thermometer, but not either of the ping pong balls. We hypothesize that this indicates ping pong balls are too sensitive to small changes in air speeds to use a physics-based model to back-calculate $t_r$ to any reasonable degree of precision. The black line is $y=x$.

These results indicate that the measured parameters can be calibrated for large globes, but for small globes the physics-driven empirical model breaks down. We can confirm this by studying the *Ri* number that indicates flow regime prevalence, where forced is $Ri<0.1$, mixed is $0.1<Ri<10$, and free is $10<Ri$. By plotting the standard corrected values in Fig. 7, and now visualizing the *Ri* regimes overlaid on the $t_r$ versus $t_{r.g}$ scatter, we see visible clusters in the three predicted regimes (free-, mixed-, and forced- convection dominated) for the large globe, but not for the small globes.

The ISO7726 standard correction uses a binary selection of forced or free convection parameter. The free convection (Eq. 4) is used only when it is greater than the forced convection value calculated in Eq. (5). Our experiments were designed to have a significant free convection component. However, only 4 out of 45 data points had a free convection coefficient larger than the forced convection coefficient. This discrepancy over-attributes convective effects to forced convection.

**Figure 8:**
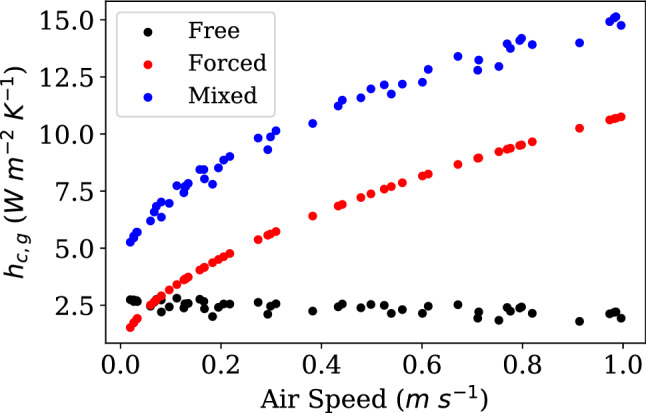
Comparison in mixed, free, and forced convection heat transfer coefficients for standard globe over all 45 experiments. Force convection is only dependent on velocity so it creates a smooth curve. The free and mixed models include dependency on $t_a-t_g$, which generates the variation. The mixed model combines effects of both and converges to the free model at $v_a$ goes to zero and approaches the forced model as it increases.

Our proposed mixed convection heat transfer correction is shown in Fig. 8. Interestingly, it is always larger than the sum of the individual free and forced convection components. This implies that for the studied geometry, a globe thermometer will lose relatively significant amounts of heat to both free and forced convection. As the forced convection signal increases relative to the mixed convection sum, the proportional difference diminishes, implying this can be a universally applicable model. As the mixed convection relationship in Eq. (11) has velocity go to zero it becomes the free convection model. As velocity increases, it drives $t_a-t_g$ down and eventually overrides free convection and approximates the forced convection model.

**Figure 9:**
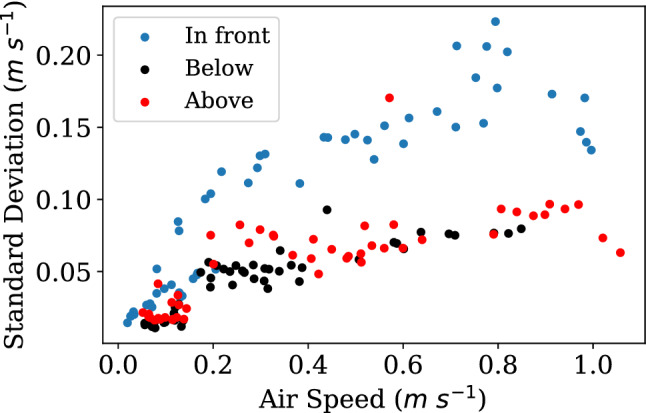
Distribution of error for each air speed sensor’s measurements, positioned ‘In front’ of, ‘Below’, and ‘Above’ the globe thermometers. Air speed measurements taken in front of the sensor were used for each analysis, as the mean values produced the highest fidelity results using the mixed convection model, despite the largest relative errors.

The air speed versus standard deviation of each air speed measurement from all three probes is shown in Fig. 9. Measuring the air speed 25 cm in front of the globes had the highest degree of variability compared to the sensors positioned above and below. Despite the larger uncertainty, air measurements taken from in front of the globe were used in all other plots in this paper for reasons outlined in the *Discussion* section.

**Figure 10:**
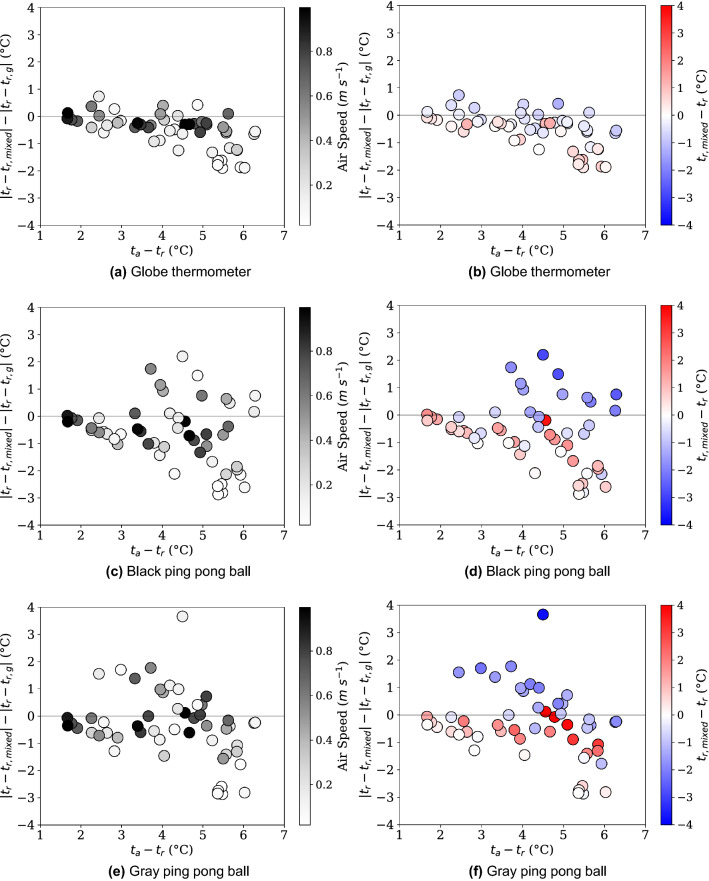
A relative comparison between the standard correction factors and the proposed mixed correction factor is shown for each globe thermometer. Gradients correspond to air speed (left and grayscale) and absolute error (right and blue-to-red color).

In addition, absolute comparisons of discrepancies between the ISO7726 method and our proposed mixed convection correction are shown in Fig. 10. There are two coloration versions provided, indicating the air speed and the absolute error associated with each data point. In these figures, axes have been normalized across all figures for true comparison.

In general, errors are smallest for the large diameter globe, however the difference is largest as air speed decreases while the temperature difference between the air and surroundings increases (as $t_a-t_r$ increases). Visually, the $t_a-t_r$ difference combined with $v_a$ are good indicators of the difference between the two models across all globe diameters. This is indicative that free convection effects are not only neglected typically, but are also difficult to accurately account for.

**Figure 11:**
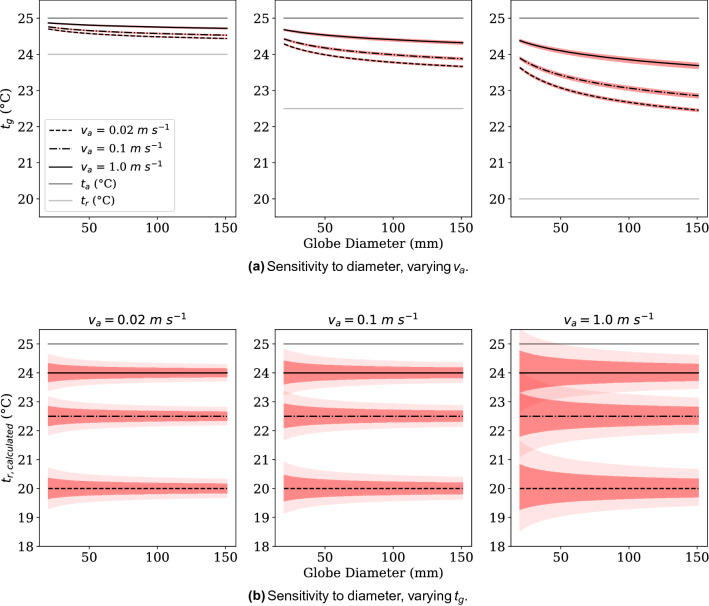
Discrete sensitivity analysis for different constant conditions for $T_g$ and *D*. In (**a**), air speed was varied by $-$2 to 2 standard deviations, based on our observed measurement range. $t_r$ and $t_a$ were fixed quantities, and the resulting range of $t_g$ was back-calculated from our proposed mixed convection correction factor. This was done for three average air speeds, producing a narrow band of expected $t_g$ fluctuations. Figure (**b**), however, allows both $t_g$ and the coincident $v_a$ to vary as was observed in our measurements, from $-$2 to 2 standard deviations again. The resulting $t_r$ value varies greatly, particularly at low air speeds. this demonstrates the importance of ensuring steady state.

A simple sensitivity analysis was conducted to see how error propagated through the mixed convection correction looking at the influence of variability in measured air speed and globe temperature on radiant temperature results. One and two standard deviations from collected data for air speed and globe temperature are used. The two standard deviations create the dark and lighter shading regions in Fig. 11. Data is plotted over a variable globe thermometer diameter to demonstrate the ranges of the different sensors used.

Figure 11a plots the predicted fluctuation of the globe thermometer temperature due to air speed fluctuations at different globe diameters. This is plotted for three different radiant temperature conditions. This shows the error based on the standard deviation of our measured air speeds and represents the intrinsic variation that would be predicted to occur at the globe thermometer. Though the variation bands plotted are narrow, those small variations have significant impacts as they would be magnified in practice to determine the radiant temperature, $t_r$. As the radiant temperature separates from the air temperature ($t_r-t_a$ increases), the smaller diameter diameter globes remain closer to the air temperature. Therefore when the corrected radiant temperature would be calculated that error would be magnified as $t_g-t_a$ has to be used to determine the much larger $t_r-t_a$.

In addition to the intrinsic error caused by air speed that would propagate to the corrected radiant temperature, Fig. 11a shows the direct propagation of error from the velocity measurements radiant temperature correction calculation. The ranges plotted are based again on the standard deviation of our measured air speeds for three different experimental air speeds. For the higher air velocity of 1 m/s the small 40mm diameter globe the variation is significant. While the error is always within the ISO standard acceptability limits of +/$-$ 5 $^{\circ }$C, it is clear that extrapolating data from smaller globe thermometers has a larger error potential. These errors depicted from our air velocity are also still lower than the variation we observed, particularly for the small globes. The errors here do not include the influence of sensor position relating to whether air speeds measured represent the real convective flow at the globe. It is also not indicative of the potential mismatch in the response time of the air speed sensor, and whether the standard 2-min time average is representative of the actual average convective exchange that occurred on the globe.

## Discussion

The results clearly demonstrate a significant source of error when convection is not properly accounted for around the globe thermometer, the most common mean radiant temperature measurement tool. The error with the standard 6 inch globe implies that many measurements taken should be revisited for air velocities below 1 m/s when the measured globe temperature is significantly different than the air temperature. The error with the 40mm "ping pong" ball globe shows that these devices are not capable of accurately predicting the radiant heat transfer without extremely precise control and measurement of complex free and mixed convection conditions.

There is a steady state requirement that is difficult to achieve with any acceptable degree of certainty during mixed convection operating regimes that makes small diameter globes poorly performing sensors when accuracy within 5 $^{\circ }$C is required. Likewise, for coincident air speed measurements it’s important to measure prior to the air interacting with the globe, but this is not always possible in real-world settings with inconsistent air sources.

Saliently, nowhere in current standards or literature is there a recommendation for where to measure air speed relative to the globe. We described the direct error in our measurements in Fig. 11, but there are many other sources of bias from procedural variation in air speed measurement such as air speed sensor placement. Our air speed data measured above and below the globe thermometers have less noise than those measured in front of the globe thermometer, as can be seen in Fig. 9. Yet when comparing to a ground truth value, the more consistent, less noisy above and below values produces less accurate mean radiant temperature values. In general, measuring very low air speeds for just free convection that might be observed above and below the globe is technically very difficult, though future work could use more advanced tools to confirm some of our hypotheses. We presume that the globe itself impacts air flow in the flow path for the sensors positioned above and below, slowing and adding stability to the vertical vector components it measures related. The sensor in front would measure a potentially more turbulent field generated by the fans. While more variable, the sensor in front would measure a field more representative of the forced convection impinging upon the globe. Yet despite the low standard deviations, when using either the above or below air speeds in correction equations compared to values from in front, lower-fidelity values were produced relative to the measured ground truth value for both the standard and mixed convection equations. In addition to inconsistencies in the position of the air speed sensor, there are potential mismatches in the response time of the globe to changes in air speed and convection. Standard practice is to take a 2-min average, but clearly globes of different sizes, materials, and masses will all have different response times to changes in conditions^14,53^. A simple time-average of the air speed may also not be representative of the average convection occurring at the globe over time. In summary, beyond the detailed measurements we have made of globe performance, there are further considerations for how convection affects radiant temperatures derived from globe thermometers.

These results have major implications for how thermal comfort studies have been carried out, the reported precision of results, and the best practices going forward. We have proposed a mixed convection correction procedure for the standard globe that seems to perform better. Moreover, we have demonstrated how simple pyrgeometers and non-contacting surface temperature sensors that are readily available can be used to directly measure radiant heat transfer. Ironically, these were the first tools used to validate the globe thermometer in the 1930’s, which subsequently were replaced by simple globe thermometers. This was in a time when complex analog electronic components were expensive. We believe modern electronic devices are now so inexpensive that it is appropriate to rethink whether multiple devices or the use of thermal imaging cameras can enable a better measurement of radiant heat transfer in space than the common globe thermometer. We have proposed several such devices in our previous work measuring radiant environments^54–56^.

The majority of contemporary research still uses the globe thermometer to determine the MRT, but we have found little literature discussing these physical convective errors generated at the measurement device. This could be due in part to the fact that the device is meant to measure a physical quantity unrelated to convection, and thus the influence of convection is not questioned during the measurement.

The findings also provide lessons to be learned in the general acceptance of measurement techniques and analysis methods as standard practice. When methods are replicated, and the procedures simply cited without considering the implications of the underlying physics, persistent error and bias can be generated. In this case it became more common to simply cite the method, and then report the thermistor resolution as the device precision rather than consider the physics of the globe thermometer measurement. In order to compare our work to the literature we found that the most relevant sources of discussion around the corrections for the globe thermometer occurred when the original device was invented and when it was refined to use the smaller globe. Therefore, instead of a limited comparison to contemporary work in our discussion, we have conducted a longer historical review spanning that time-frame leading up to a discussion, review, and comparison of our work to current practices.

## Conclusions

We have demonstrated significant sources of potential error in the use of globe thermometers (a ubiquitous tool for analyzing comfort in buildings) to measure mean radiant temperature. These are caused by inappropriate use of convective exchange corrections applied to the measured globe temperature over the past 30 years. Using a laboratory with highly controlled surface temperatures and a set of precise pyrgeometers and air velocity sensors, we demonstrate that standard corrections do not adequately capture the relationship between the globe temperature and the true mean radiant temperature for low air speeds where mixed and free convection dominate the exchange. We propose a unified method for properly accounting for free and forced convection simultaneously, and demonstrate that there is a potential for consequences associated with this systematic error across the thermal comfort domain. But perhaps most noteworthy, we provide a sensitivity analysis to demonstrate that uncertainty in mean radiant temperatures is proportional to globe diameter, which precludes small diameter sensors from high-precision measurement within 2 $^{\circ }$C.

### Future Work

In this study, high-precision pyrgeometers were used to measure the mean radiant temperature. However, cheaper, digital-output thermopiles could be used to achieve convection-insensitive measurements. The authors use these in other research, yet the method has not been explicitly validated. Future work should also focus on explaining which factors of mean radiant temperature are most important to capture, essentially to answer the question of whether accurately knowing the mean radiant temperature as currently defined for a point in space is sufficient to accurately predict the human body’s radiant exchange with its surroundings. Further refinements can be made to the definition of ’mixed convection’ and limits of the application of the n-parameter method described in Eq. (11).

## Materials and Methods

### Apparatus

The measurements were conducted in the climate chamber of the Department of Building Physics at the Bauhaus-University Weimar. The chamber is a 3 $\times$ 3 $\times$ 2.44 m room situated in a 5.40 $\times$ 5.40 $\times$ 3.05 m laboratory hall to keep it isolated from the outdoor environment. The chamber is tempered by water-bearing capillary tubing placed under the finishing layer (tiles for the floor and gypsum plaster for walls and ceiling). The chamber door is not tempered, yet due to its low overall heat transfer coefficient ($U = 0.29 ~W/m^2K$) and the rather small air temperature difference between the two sides of the door, the non-tempered door does not strongly impact the air or surface temperatures in the chamber. The chamber implements a ventilation system to introduce fresh or recirculated air in an adjustable flow rate into the space. The temperature in the chamber can be defined by controlling the temperature of each surface separately or the temperature of the ventilation system, or both.

Negative temperature coefficient thermistors (NTC) with an uncertainty of $\pm 0.2\, ^{\circ }$C and a resolution of 0.01 $^{\circ }$C were used to monitor air and surface temperature during the experiments. To measure air velocity, omni-directional hot-wire anemometers with an accuracy of $\pm 1.5\%$ of the measured value and a resolution of 0.001 m/s were utilized. Relative humidity in the chamber was constantly monitored using a digital humidity sensor with an accuracy of $\pm 2\%$ of the measured value (at $25 ^{\circ }\hbox {C}$). For thermography, FLIR B400 infrared camera with an accuracy of $\pm 2 ^{\circ }\hbox {C}$, an image resolution of 320 x 240 pixels, and a measurement resolution of 0.1 $^{\circ }$C was used. Since the climate chamber is relatively small, a wide-angle lens of $45^{\circ }$ was used with the camera to allow capturing a wide field of view.

To evaluate the errors of the globe thermometer, a standard 150-mm globe along with a gray and a black ping-pong ball globes (D = 40 mm) was used. The standard black globe thermometer (D = 150 mm) was equipped with a Pt100 element with an accuracy of $(0.3 + 0.005|\hbox {T}|)\, ^{\circ }\hbox {C}$ of the measured value and a resolution of 0.01 $^{\circ }$C. The two ping pong balls were constructed with standard plastic painted gray and black, respectively. Inside each was a high-precision epoxy thermistor ($\pm 1 ^{\circ }\hbox {C}$). The values recorded by the globe thermometers were evaluated against the radiometric ground-truth mean radiant temperature measured by six pyrgeometers mounted on the faces of a 44 mm wooden cube. More on the justificiation of a radiometric ground-truth reading is presented in Fig. 13 and the corresponding section. The pyrgeometers were all Apogee SL-510-SS devices (± 0.3 $^\circ$ C or 0.12 mV per *W* $m^{-2}$). The emissivity of the black and gray ping pong balls within the 8-15 micron region was confirmed experimentally using a FTIR spectrometer (Nicolet i8) to be the same, we used both since the literature contains examples of both black and gray ping pong balls^57^.

All the implemented sensors were controlled remotely from the outside the chamber to avoid disturbing the measurements.

### Experimental configuration

**Figure 12:**
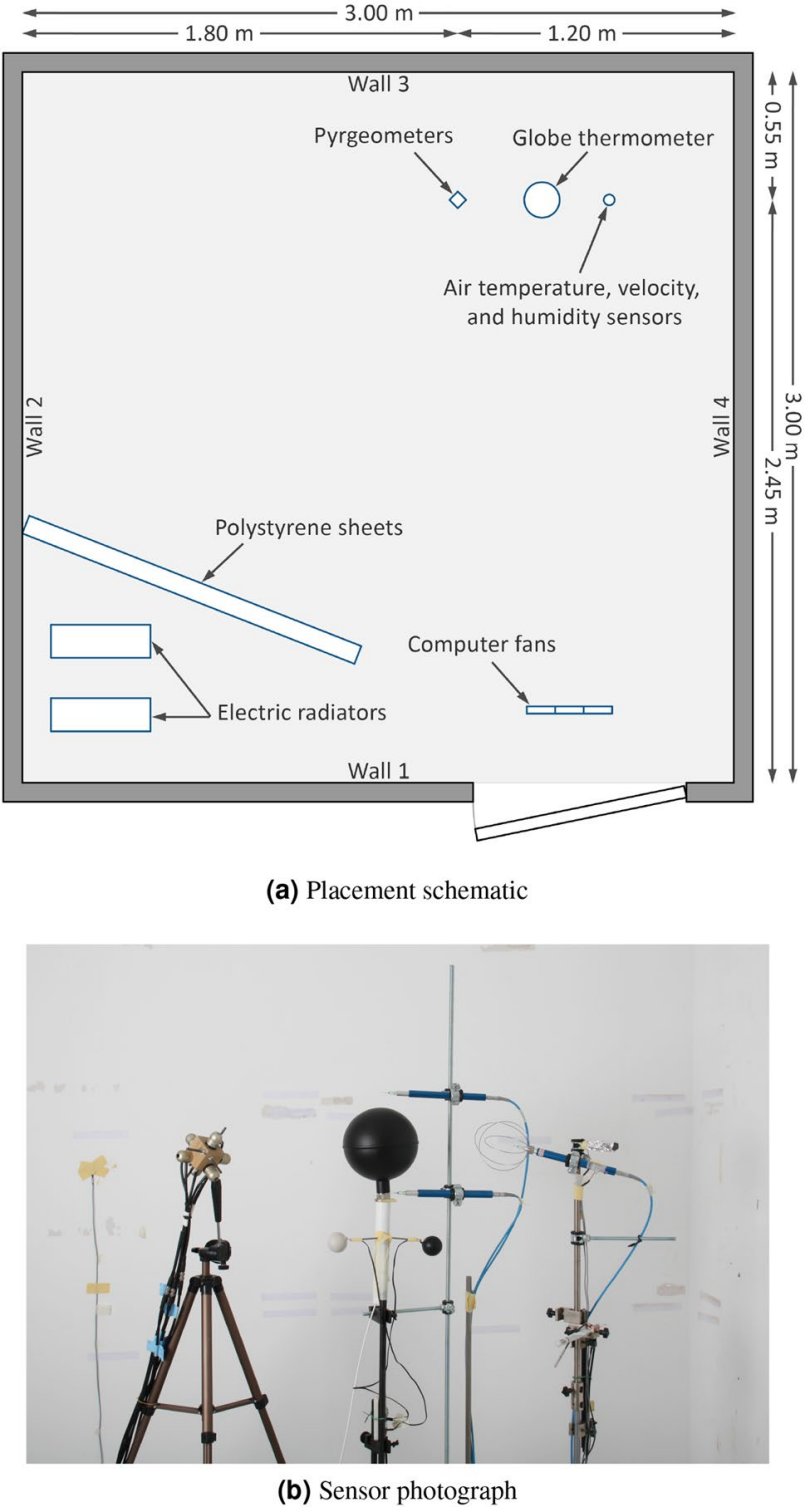
The experimental configuration in the chamber including the 6-sided ‘ground truth’ pyrgeometer measurement device on the wooden cube,large globe, gray and black ping pong balls, and air temperature and velocity probes.

The implemented sensors were mounted on stands near the corner of the chamber (Fig. 12). An air velocity probe was mounted near the standard globe (at 25 cm to the centre of the globe) to measure the room air velocity. This sensor had a fine response time of 100 ms. Two additional velocity probes were mounted directly below and above the globe to investigate the ascending and descending convective flow; the distance between the tip of the velocity probe and the surface of the globe was 3 cm. These sensors had a smoothed response time of 2 s. Two air temperature sensors were used to monitor room temperature: one shielded with aluminium foil and one without foil. Both sensors were mounted at the base of the room air velocity probe. Data from the shielded sensor was used in the analysis. Seven NTC sensors were used to monitor the surface temperature (4 walls, ceiling, floor, and door). The surface temperature sensors were mounted at the centre of each surface. The height of the standard globe, the pyrgeometers cube, the room velocity probe, and room air temperature was 128.5 cm (from the floor to the centre of the sensor).

To create a large separation between surface temperature and air temperature, two electric heaters (1900 W each) were placed in the chamber to heat the air while cooling the walls, floor, and ceiling of the chamber. To prevent impacting the measured mean radiant temperature, the heaters were vertically shielded behind 80-mm expanded polystyrene insulation sheets fitted with aluminium foil sheets to reflect radiation. Different degrees of temperature separation were created by controlling the operation capacity of the electric heaters and the number of implemented heaters. This resulted in a surface temperature range of 16.1–20.4 $^{\circ }$C, an interior air temperature range of 20.4–28.0 $^{\circ }$C, and a mean radiant temperature range of 18.2–23.0 $^{\circ }$C. The range of air to surface temperature difference was 1.7–6.3 $^{\circ }$C measured during a total of 52 experimental boundary conditions. 7 of the 52 were redundant cases, so the unique 45 are presented in this manuscript for clarity. Air movement was induced in the chamber by controlling the ventilation system. Additionally, a cluster of 9 computer axial fans (D = 120 mm) was used to generate a relatively homogeneous airflow directed at the sensors. This allowed a finer control of the target air velocity which ranged from 0.02–1.00 m/s at 25 cm from the centre of the standard globe thermometer.

The measurements were conducted under a steady state which was achieved by giving the chamber enough leading before each set of boundary conditions. The criterion for steady state was defined as globe-measured fluctuations within 0.5 $^{\circ }$C. Data was collected for each set of boundary conditions for 30 minutes with a sampling interval of 1 s.

### Geometric justification of ground truth measurement

**Figure 13:**
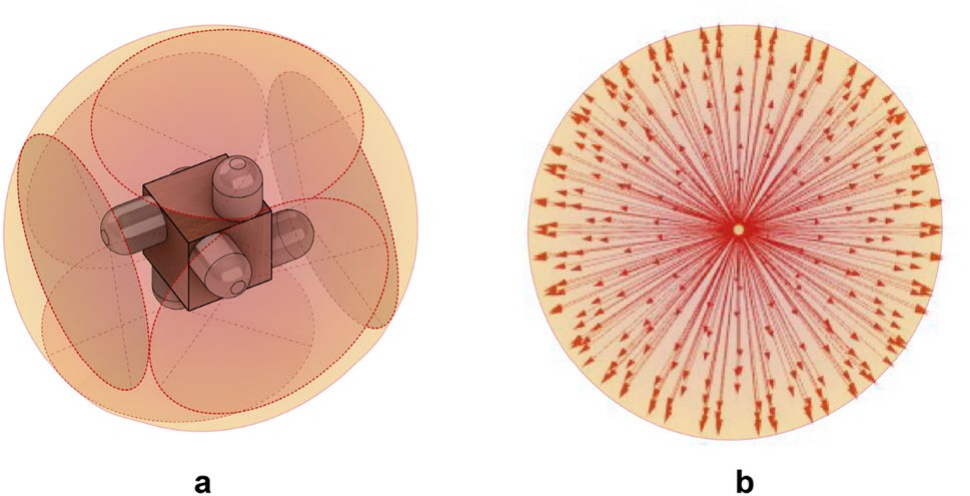
(**a**) 6-pyrgeometer arrangement around a cube to cover the sphere of view from a point in space; (**b**) visualization of the vector-based simulation of MRT used for validation of the ground-truth measurement device.

Our ground-truth measurement device consists of 6 pyrgeometers. The arrangement is based on the ASHRAE55 adapted standard from ISO7726 (International Organization for Standardization 1998) for calculation of MRT based on multiple-plane radiant temperature. This method is described at length in Thorsson et. al 2007, as the most accurate way of determining outdoor MRT. This 6 directional arrangement provides a planar irradiance reading in each of the directions: up, down, left, right, front back, and the MRT is calculated as a weighted average of these planar measurements. The 6 pyrgeometers in the device we constructed for indoor MRT measurement are aranged around a small cube to provide a full sphere of view from an arrangement that is as close-as possible to a single point in a room, shown in Fig. 13a.

We tested the accuracy of this device using a vector-based simulation discussed in Aviv et al.^58^. The simulation includes 1280 vectors, which are generated by the level of geodesic subdivision of a sphere originating from the same point in the room where the measurement device was centered, Fig. 13b. The intersections between the vectors and the room’s surfaces are used to calculate the MRT at that point based on temperature readings and view factor of each surface. We compared the simulation results to the readings from the device and they have been within 0.3 $^{\circ }$C from each other, which indicates a high degree of accuracy. Additionally, we checked the inherent potential error in the pyrgeometer array caused by overlaps in the ranges of the 6 pyrgeometers. The overlaps may cause different MRT readings for different orientations of the device in space. However, a maximum difference of up to 0.08 $^{\circ }$C was detected within the chamber setup conditions when simulating a change in the device’s orientation multiple times which we can consider negligible.

### Data processing and error analysis

The process to extract the mean radiant temperature, $t_r$ from a globe thermometers require air temperature, $t_a$, globe thermometer temperature, $t_g$, and air speed, $v_a$, to be measured. The mean radiant temperature was calculated from $t_g$ from a standard *D* = 150 mm black globe (Fig. 5a), *D*=40 mm black ping pong ball (Fig. 5b), and 40 mm gray ping pong ball (Fig. 5c) according to the ASHRAE standard given in Eq. (2). $\varepsilon$ was set to 0.95 for all three sensors, confirmed to be within 1% of the true value in our previous work^59^. A constant hemispherical emissivity is justified since the sensors were not subject to solar radiation in the test environment, only longwave thermal radiation.

2 $$\begin{aligned} t_{r,g} = \root 4 \of {(t_g+273.15)^4-\frac{1.1 \cdot 10^8 \cdot v_a^{0.6}}{\varepsilon \cdot D^{0.4}}(t_a-t_g)}-273.15 \end{aligned}$$

By inspection, this equation reduces to $t_r=t_g$ when $v_a$=0, implying that effects due to free convection are negligible. In the ISO7726 standard, there is a generalised method for account for free convection. The ISO7726 method begins with Eq. (3), which allows for substitution of any convective heat transfer coefficient, $h_{cg}$. Here, $\sigma$ is the Stefan-Boltzmann constant.

3 $$\begin{aligned} t_{r,g} = \root 4 \of {(t_g+273.15)^4-\frac{h_{cg}}{\varepsilon \sigma }(t_a-t_g)}-273.15 \end{aligned}$$

The first step in the ISO method is to determine whether $h_{cg}$ is larger when using their free convection correlation, given in Eq. (4), or when using their forced convection correlation, given in Eq. (5).

4 $$\begin{aligned} h_{cg,free}= & {} 1.4 \root 4 \of {\frac{t_a-t_g}{D}} \end{aligned}$$

5 $$\begin{aligned} h_{cg,forced}= & {} 6.3\frac{v_a^{0.6}}{D^{0.4}} \end{aligned}$$

Once the determination for which is larger is made, the respective correlation for $h_{cg}$ is chosen. For all data collected in this experiment, 4 out of 45 points have a larger free convection coefficient than forced.

When using the forced convection correlation equation for $h_{cg}$, one arrives at the ASHRAE equation. Meaning ASHRAE systematically neglects free convection, whereas the ISO7726 standard has a pathway for selection.

In our previous publication highlighting the potential for significant errors in both the ISO7726 and ASHRAE methods when mixed convection is not accounted for^19^, however the finding was an unexpected aside from the main experiment and the outdoor environment was too uncontrolled to provide meaningful recommendations.

In this paper, we provide a validation for a unified mechanism for mixed-mode convective heat transfer that we believe is a better description of the steady state heat transfer a globe thermometer experiences than both the current ISO7726 and ASHRAE standards reflect. This empirical model goes one step back to the Nusselt number, *Nu*, correlations for spherical bodies in air experiencing both free and forced convection, Eqs. (6) and (7), respectively. In these equations, *Re*, *Ra*, and *Pr*, are the Reynolds, Rayleigh, and Prandtl numbers, and equations for these variables are provided in Eqs. (8), (9), and (10). The equations are all valid in the operating regime for this experiment.

6 $$\begin{aligned} Nu_{free}= & {} 2+\frac{0.589Ra_D^{\frac{1}{4}}}{(1+(0.469/Pr)^{\frac{9}{16}})^{\frac{4}{9}}} \end{aligned}$$

7 $$\begin{aligned} Nu_{forced}= & {} 2+(0.4Re^{\frac{1}{2}}+0.06Re^{\frac{2}{3}})Pr^{0.4} \end{aligned}$$

8 $$\begin{aligned} Re= & {} \frac{v_aD}{\nu } \end{aligned}$$

9 $$\begin{aligned} Ra= & {} \frac{g \beta }{\nu \alpha }(t_a-t_g)D^3 \end{aligned}$$

10 $$\begin{aligned} Pr= & {} \frac{c_p \mu }{k} \end{aligned}$$

Equations (6) and (7) are always used to produce values for $h_{cg}$ regardless of the air speed^60^. A weighting mechanism from^61^ is then used to determine the relative importance of the different coefficients. The weighting mechanism is shown in Eq. (11), and values for *n* were empirically derived from our current work for each globe thermometer, varying *n* to minimize error to the ground truth measurement. Empirical values do not exist for this specific problem of a radiantly forced, but otherwise isolated sphere.

11 $$\begin{aligned} Nu = \frac{h_cD}{k}; h_{cg,weighted}=\frac{k({h_{cg,free}^n+h_{cg,forced}^n})^{\frac{1}{n}}}{D} \end{aligned}$$

Without weighting, Eqs. (5) and (4) do not produce the same values for *Nu* as produced with Eqs. (7) and (6). These discrepancies are due to the evolution of the empirical models used to represent complex free and forced convection phenomena. Future work should consider parallel research in the fluid dynamics domain to where these convection parameters are researched and defined.

Further dimensional analysis is provided here for using mixed convection as the heat balance calculation method. Just as often free convection problems are described by the Grashof number *Gr*, and the Reynolds number, *Re*, describes laminar flow in forced convection dominated problems, the Richardson number, *Ri*, describes mixed convection regimes. In general, a value of $0.1<Ri<10$ means that mixed convection is the dominant mode, with any number above 10 meaning that free convection dominates, and any number below 0.1 means that forced convection dominates. *Ri* is defined in Eq. (12), where *g* is acceleration due to gravity and $\beta$ is the thermal expansion coefficient.

12 $$\begin{aligned} Ri=\frac{Gr}{Re^2}=\frac{g \beta (t_a-t_g)D}{v_a^2} \end{aligned}$$

It is cautioned that *Ri* is only an indicator of mixed convection, since mixed convection situations are unique. However, *Ri* is a convenient metric to portray the relative impact of free and forced convection.

A linear regression was also performed using a least-squares method in Python, regressing the ground truth $t_r$ measurement with $(t_a-t_g)$ and $v_a$ as the two independent variables. This was done as a method of assessing whether the physics-based empirical model performed better than a very simple, potential driven heat transfer regression model.

## List of symbols

$$\alpha$$: Thermal diffusivity
$\beta$: Thermal expansion coefficient
$$\mu$$: Dynamic viscosity
$$\nu$$: Kinematic viscosity
$\sigma$: Stefan-Boltzmann constant
$$\varepsilon$$: Surface emissivity
*c*: Specific heat capacity
*D*: Globe thermometer diameter
*g*: Acceleration due to gravity
*Gr*: Grashoff number
*h*: Heat transfer coefficient
*k*: Thermal conductivity
*n*: Weighting factor for mixed convection
*Nu*: Nusselt number
*Pr*: Prandtl number
*Ra*: Rayleigh number
*Re*: Reynolds number
*Ri*: Richardson number
*t*: Temperature, $$^{\circ }$$C
$_a$: Value for air
$_r$: Radiant value as measured by pyrgeometers
$_{c,weighted}$: Weighted mixed convection heat transfer coefficient
$_{calculated}$: Referring to any method of calculating the mean radiant temperature for comparison to the ground truth measurement
$$_{cg}$$: Convective coefficient
$_{forced}$: Referring to forced convection
$_{free}$: Referring to free convection
$_{g}$: Value measured directly from a globe
$_{p}$: Referring heat capacity at constant pressure
$_{r,g}$: Radiant value measured by a globe thermometer using standard correction
$_{r,mixed}$: Radiant value measured by globe determined by a mixed convection correction
